# Darunavir resistance spectrum in darunavir-na#239;ve patients harboring virological failure to antiretroviral therapy

**DOI:** 10.1186/1758-2652-13-S4-P133

**Published:** 2010-11-08

**Authors:** AG Marcelin, C Charpentier, M Wirden, D Descamps, V Calvez

**Affiliations:** 1 Pitie-Sapetriere Hospital, Paris, France; 2 Bichat-Claude Bernard Hospital, Paris, France; 3 Pitie-Salpetriere Hospital, Paris, France

## Purpose of the study

Darunavir is one of the protease inhibitors that is recommended to treat protease inhibitor-naïve or -experienced patients. Recent studies have determined the spectrum of darunavir activity in patients failing to antiretroviral therapy. Darunavir resistance mutations in protease gene have been identified (V11I, V32I, L33F, I47V, I50V, I54L, I54M, T74P, L76V, I84V and L89V) and allow to classify viruses as sensitive (<3 mutations), possibly resistant (3 mutations) or resistant (≥4 mutations) to darunavir.

## Methods

1583 genotypic resistance tests performed between 2008 and 2009 for darunavir-naïve patients experiencing virological failure (whatever the antireroviral combination used) were analyzed retrospectively. Protease gene was sequenced and aminoacid changes analyzed at time of virological failure. The number of darunavir resistance mutations were determined and the strains were classified regarding the spectrum of darunavir activity (ANRS algorithm V18).

## Summary of results

Among these experienced patients failing antiretroviral therapy, 63% harbored no darunavir mutations in the protease gene and 12%, 16%, 4% and 5% harbored 1, 2, 3 and at least 4 darunavir mutations, respectively. Patients with viruses harboring darunavir mutations had lower HIV-1 viral load than patients with viruses without any darunavir mutations.

## Conclusions

This study shows that the percentage of genotypic fully resistant strains to darunavir is rare in a population of darunavir-naïve patients experiencing virological failure. A large proportion of patients harbored viruses without any darunavir resistance mutations allowing the use of darunavir/r (800/100 mg) QD. Virological failures without selection of any mutations showed higher viral load level rebound probably related to lack of adherence.

